# Location of the radial nerve along the humeral shaft between the prone and lateral decubitus positions at different elbow positions

**DOI:** 10.1038/s41598-021-96458-6

**Published:** 2021-08-26

**Authors:** Sitthiphong Suwannaphisit, Wachirakorn Aonsong, Porames Suwanno, Chaiwat Chuaychoosakoon

**Affiliations:** Department of Orthopedics, Faculty of Medicine, Prince of Songkla University, 15 Karnjanavanich Road, Hat Yai, Songkhla, 90110 Thailand

**Keywords:** Musculoskeletal system, Nervous system

## Abstract

Identification of the radial nerve is important during the posterior approach to a humerus fracture. During this procedure, the patient can be placed in the prone or lateral decubitus position depending on the surgeon’s preference. The distance from the radial nerve to the osseous structures will be different in each position. The purpose of this study was to identify the safety zones in various patient and elbow flexion positions. The distances from the olecranon to the center of the radial groove and intermuscular septum and lateral epicondyle to the lateral intermuscular septum were measured using a digital Vernier caliper. The measurements were performed with cadavers in the lateral decubitus and prone positions at different elbow flexion angles. The distance from where the radial nerve crossed the posterior aspect of the humerus measured from the upper part of the olecranon to the center of the radial nerve in both positions at different elbow flexion angles varied from a mean maximum distance of 130.00 mm with the elbow in full extension in the prone position to a minimum distance of 121.01 mm with the elbow in flexion at 120° in the lateral decubitus position. The mean distance of the radial nerve from the upper olecranon to the lateral intermuscular septum varied from 107.13 to 102.22 mm. The distance from the lateral epicondyle to the lateral edge of the radial nerve varied from 119.92 to 125.38 mm. There was not significant contrast in the position of the radial nerve with osseous landmarks concerning different degrees of flexion, except for 120°, which is not significant, as this flexion angle is rarely used.

## Introduction

The incidence of fractures of the shaft of the humerus is 14.5 per 100,000 per year^1^. Surgical intervention for a humerus fracture is common, with the posterior approach mostly preferred for fractures of the mid to distal humerus. The incidence of iatrogenic injury to the radial nerve has been reported to range from 0 to 43% with different surgical approaches^2–4^. The largest retrospective study to date reported iatrogenic radial nerve palsy after fixation in 12.2% of all participants (17.9% with a posterior tricep-sparing approach and 11.7% with a posterior tricep-splitting approach). Implant placement could result in irritation or damage to the nerve^4^. Injury to the radial nerve resulting in radial neuropathy or radial nerve palsy leads to wrist drop with a poor outcome^5^. To decrease the incidence of radial nerve palsy, identification of the exact location of the radial nerve in relation to the osseous structures is essential when preparing a posterior approach to a humerus fracture.

Various studies have examined the location of the radial nerve in relation to common anatomical landmarks, the olecranon fossa^5,6^, the intersection of the long lateral head of the triceps and triceps aponeurosis^7,8^, and four fingerbreadths from the lateral epicondyle^9^. All of these studies, however, evaluated the radial nerve in only one position, which does not accurately reflect the real clinical situation in which the patient can be placed in the prone, lateral decubitus or supine position, depending on the surgeon’s preference, and the distance from the radial nerve to the osseous structures will be different in different positions.

To date, no studies have investigated the distance from the radial nerve to important landmarks in these common surgical positions, the lateral decubitus and prone positions, with the elbow flexed at different angles. Hence, the purpose of this study was to identify the safety zones of various patient and elbow flexion positions to help reduce the risk of radial nerve injuries.

## Results

**Figure 1:**
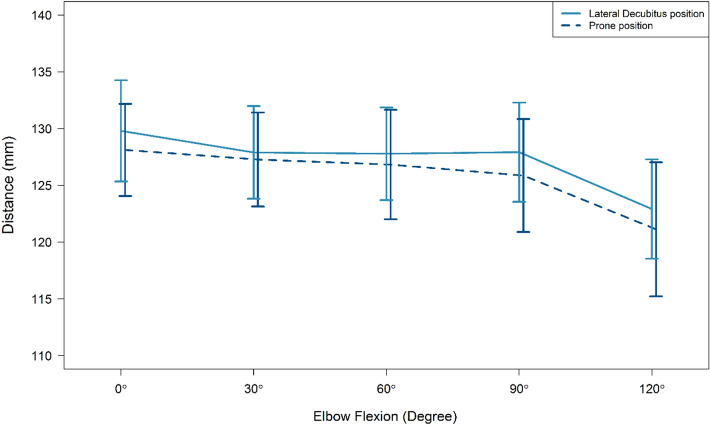
A line graph showing the distance from the upper part of the olecranon to the center of the radial nerve in each position.

The humeral length averaged 28.87 cm (± 2.71 cm, range 24.50 to 32.60 cm). The mean distances from the upper part of the olecranon to the radial nerve in the radial groove at different elbow flexion angles in the prone and lateral decubitus positions are summarized in Table 1. The mean distances from the upper part of the olecranon to the radial nerve in the lateral intermuscular septum at different elbow flexion angles in the prone and lateral decubitus positions are summarized in Table 2. The mean distances from the lateral epicondyle to the radial nerve in the lateral intermuscular septum at different elbow flexion angles in the prone and lateral decubitus positions are summarized in Table 3. From the upper part of the olecranon to the central posterior point where the radial nerve crosses the humerus, the distances were significantly lower with increasing elbow flexion angles, as shown in Fig. 1. There were no significant differences between the right and left sides of the same cadavers in terms of humeral length or distances of the radial nerve to osseous landmarks. There was excellent intraobserver correlation between the two orthopedists, with correlation coefficients between 0.77 and 0.99.

**Table 1:** Distances from the upper olecranon to the center of the radial nerve in the posterior shaft of the humerus.

Elbow flexion angle (°)	Prone position (mm) (n = 20)		Lateral decubitus (mm) (n = 20)		Mean difference (95% CI)	*P* value
	Mean ± SD	Minimum	Mean ± SD	Minimum		
0	130.00 ± 2.07 (B1)	113.70	128.00 ± 1.89 (E1)	114.90	1.69 (− 1.99–5.35)	0.35
30	128.10 ± 2.81 (B2)	114.95	127.16 ± 2.70 (E2)	112.67	0.63 (− 2.47–3.72)	0.68
60	127.98 ± 2.81 (B3)	115.18	126.72 ± 2.94 (E3)	104.33	0.95 (− 2.55–4.45)	0.58
90	128.12 ± 2.91 (B4)	111.66	125.75 ± 2.99 (E4)	105.67	2.06 (− 1.07–5.18)	0.19
120	123.12 ± 2.91 (B5)	110.14	121.01 ± 3.34 (E5)	94.69	1.80 (− 3.48–7.06)	0.449

**Table 2:** Distances from the upper olecranon to the lateral intermuscular septum.

Elbow flexion angle (°)	Prone position (mm) (n = 20)		Lateral decubitus (mm) (n = 20)		Mean difference (95% CI)	*P* value
	Mean ± SD	Minimum	Mean ± SD	Minimum		
0	106.00 ± 1.81 (C1)	90.50	105.92 ± 1.81 (F1)	92.43	1.21 (− 4.02–1.60)	0.38
30	107.13 ± 2.56 (C2)	91.31	107.05 ± 2.56 (F2)	93.95	1.76 (0.04–3.48)	0.40
60	103.79 ± 2.49 (C3)	88.74	103.71 ± 2.49 (F3)	91.28	3.03 (− 5.69–0.37)	0.20
90	105.78 ± 2.47 (C4)	91.4	105.69 ± 2.47 (F4)	92.25	0.58 (− 2.43–3.59)	0.69
120	102.30 ± 2.83 (C5)	86.10	102.22 ± 2.83 (F5)	86.10	1.85 (− 0.13–3.83)	0.06

**Table 3:** Distances from the lateral epicondyle to the lateral intermuscular septum.

Elbow flexion angle (°)	Prone position (mm) (n = 20)		Lateral decubitus (mm) (n = 20)		Mean difference (95% CI)	*P* value
	Mean ± SD	Minimum	Mean ± SD	Minimum		
0	122.00 ± 2.33 (D1)	102.27	122.00 ± 2.37 (G1)	104.40	0.65 (− 2.80–1.50)	0.53
30	123.21 ± 3.47 (D2)	102.29	124.94 ± 3.43 (G2)	107.74	2.37 (− 5.60–0.85)	0.14
60	124.55 ± 3.20 (D3)	106.45	125.08 ± 3.48 (G3)	105.09	1.19 (− 4.25–1.88)	0.43
90	125.38 ± 3.27 (D4)	108.01	124.24 ± 3.41 (G4)	108.34	0.50 (− 2.47–3.45)	0.73
120	119.92 ± 2.94 (D5)	105.14	124.71 ± 3.39 (G5)	108.33	5.44 (− 9.85–1.03)	0.02

The important finding in this study was that the different distances from where the radial nerve crossed the posterior aspect of the humerus measured from the upper part of the olecranon to the center of the radial nerve in both positions with different elbow flexion angles varied from a mean maximum distance of 130.00 mm with the elbow in full extension in the prone position to the minimum distance of 121.01 mm with the elbow in flexion at 120° in the lateral decubitus position. For the lateral intermuscular septum point, the mean distance to the radial nerve varied from 102.22 to 107.13 mm. The distance from the lateral epicondyle to the lateral edge of the radial nerve ranged from 119.92 to 125.38 mm.

## Discussion

This study describes the radial nerve anatomy at the posterior aspect of the humeral shaft in two different positions, the prone position and the lateral decubitus position, with various elbow angles. The distance of the nerve to the upper olecranon fossa at the center of the humeral shaft to the lateral edge at the intermuscular septum level in both positions decreased with increasing elbow flexion angles. The highlight of this study ensures orthopedic surgeons that there is very little change in the measured distance between the radial nerve and osseous landmarks, except for elbow flexion at 120°, a position that is rarely used with a posterior approach to the elbow. Therefore, during the orthopedic procedure, if the range of motion (ROM) is limited to 0 to 90° of elbow flexion, the variation is less than 3 mm and not significant.

Previous anatomic studies have measured the distance from the radial nerve to various landmarks. Artico M et al. performed a cadaveric study with the bodies placed in the lateral decubitus position with the arms adducted and the forearms completely supinated with 45° flexion of the elbow joint^10^. The mean distance between the point of the nerve crossing the lateral border of the shaft of the humerus and lateral epicondyles was 125 ± 1.3 mm. Gus and Ostrum^11^ reported that the radial nerve was located 126 ± 11 mm proximal to the lateral epicondyle at the lateral border of the humeral shaft measured in full elbow extension^11^. A recent study by Jain et al. investigated the location of the radial nerve in relation to the medial and lateral epicondyles and found that the mean distance from the lateral epicondyle to the entry of the radial nerve into the spiral groove was 113.4 ± 41 mm^12^. Similarly, our distances in the same landmarks were 123.21 ± 3.47 mm and 124.55 ± 3.20 mm at elbow flexion angles of 30° and 60°, respectively. A study by Hackl on 100 cadavers reported that the average distances from the proximal edge of the olecranon fossa to the radial nerve at the center and lateral edge of the posterior aspect of the humeral shaft were 12.7 ± 1.6 cm and 10.6 ± 1.3 cm^6^, respectively, with results similar to our results of 12.3–13.0 cm and 10.2–10.7 cm, respectively. Our results confirm that there are no significant differences in the measurements and no significant differences in the distances of the radial nerve at an elbow flexion angle of 120° compared with the numerical values from the same landmarks from previous studies measured in the static position.

In fact, the radial nerve can vary in position during upper extremity motions. One study^12^ found that shoulder abduction from 30° to 110° required 4 mm of radial nerve excursion at the elbow. The greatest radial nerve migration was 11.4 mm, with shoulder abduction at 110° and elbow flexion at 90°^13^. However, this study investigated radial nerve excursion at a point approximately 4–6 cm proximal to the lateral epicondyle, and the findings are not relevant in the situation of a midshaft humerus fracture. Another study^14^ suggested that radial nerve excursion can be improved with elbow flexion and lateral intermuscular septum release by approximately 3 times. Our current study aimed to provide accurate information concerning the points of entry and exit of the radial nerve around the elbow, which can be applied in the case of a humerus fracture. We found that there was little change in nerve location between the two positions in different elbow flexions from 0° to 90°, except when the flexion angle exceeded 120°, when the distance was ± 3 mm from the midpoint of the humerus to the lateral intermuscular septum. The reason for the small change in radial nerve distance due to its anatomy is that the course of the radial nerve runs posterior to the spiral groove and exits anteriorly via the lateral intermuscular septum, with the course usually tethering the distal part of the radial nerve of the arm, consequently resulting in minimal dynamic changes.

Surgeons can use our findings concerning the safe zone in different elbow flexion angles in their preferred position. There was minimal variation in the position of the radial nerve in the mid to distal third of the humerus. The danger zone from the lateral epicondyle to the radial nerve passing to the lateral intermuscular septum was between 122 and 125 mm at full elbow extension (90° of flexion), not at 120°, where the radial nerve distance decreased to 119 mm. Therefore, pin placement for external fixation or lateral plate fixation is relatively safe in a zone between the lateral epicondyle and 119 mm proximal to this point. The posterior approach in either the prone or lateral decubitus position can be used in diaphyseal middle-third to distal-third procedures^15,16^. In the case of a distal humerus fracture with an intact olecranon fossa or a simple fracture of the distal humerus according to the AO classifications (AO-OTA 13 types A, B and C1)^17^, we found that the olecranon fossa was a reliable landmark that could be used to estimate the location of the radial nerve when using the posterior approach^6^. Distal fixation remains dangerous, varying between 123 and 130 mm measured from the upper olecranon to the center of the radial nerve in the posterior shaft of the humerus and between 102 and 107 measured from the upper olecranon to the lateral intermuscular septum according to the degree of elbow flexion. We therefore advocate careful blunt dissection at these dangerous zones to identify the radial nerve to thus decrease the risk of iatrogenic radial nerve injury during orthopedic plate and nail fixation.

This study had potential limitation. The length of the nerve in the lateral intermuscular bundle may have been changed by dissecting the triceps muscle, although we attempted to minimize this possible problem by dissecting only the triceps and identifying and leaving the radial nerve intact. Another potential confounding effect was the sex of cadavers, which was predominantly male in our study. Further studies can be designed to decrease this potential confounding effect.

In conclusion, there was not significant contrast in the position of the radial nerve with osseous landmarks concerning different degrees of flexion. Understanding the distance of the radial nerve in both the lateral decubitus and prone positions from these common landmarks in the various common operative positions is helpful for avoiding iatrogenic radial nerve injury during a surgical approach to the mid to distal third of the shaft of the humerus.

## Methods

### Specimen preparation and study participants

Ten fresh-frozen cadavers (8 males and 2 females) with twenty upper limbs from the Faculty of Science, Prince of Songkla University, were acquired for this study. This study was carried out in accordance with the guidelines and local regulations that all the researchers must perform following the principles of universal precautions. All specimens were screened for blood-borne diseases, such as human immunodeficiency virus, hepatitis B virus or hepatitis C virus, and stored at − 20 °C. All cadavers were thawed at room temperature twelve hours before dissection. Approval from the Research and Development Office from the local Prince of Songkla University was granted for this experimental protocol (institutional review board number 63-013-11-1). The donors were not required to sign a statement about this specific study, but they did express their consent to anonymously give part of their body to science for ethically approved experiments. The mean age at death was 74.10 ± 5.74 years, and no specimens showed evidence of a history of arm surgery. There were no significant anatomical variations and no evidence of previous traumatic upper extremity injuries in any of the cadaveric arms and elbows.

### Gross anatomical dissection

The upper arms of all cadavers were dissected using a standardized protocol by a single experienced surgeon using a triceps-splitting approach. The skin and subcutaneous tissues were dissected starting with a midline incision from the posterior acromion to the olecranon. The measurements were made using a digital Vernier caliper with a precision of 0.01 mm (Insize Co., Ltd., Suzhou New District, China). The upper part of the olecranon and the radial nerve in the spiral groove and intermuscular septum where the radial nerve passes from the posterior to the lateral part of the arm were identified.

### Measurement method

**Figure 2:**
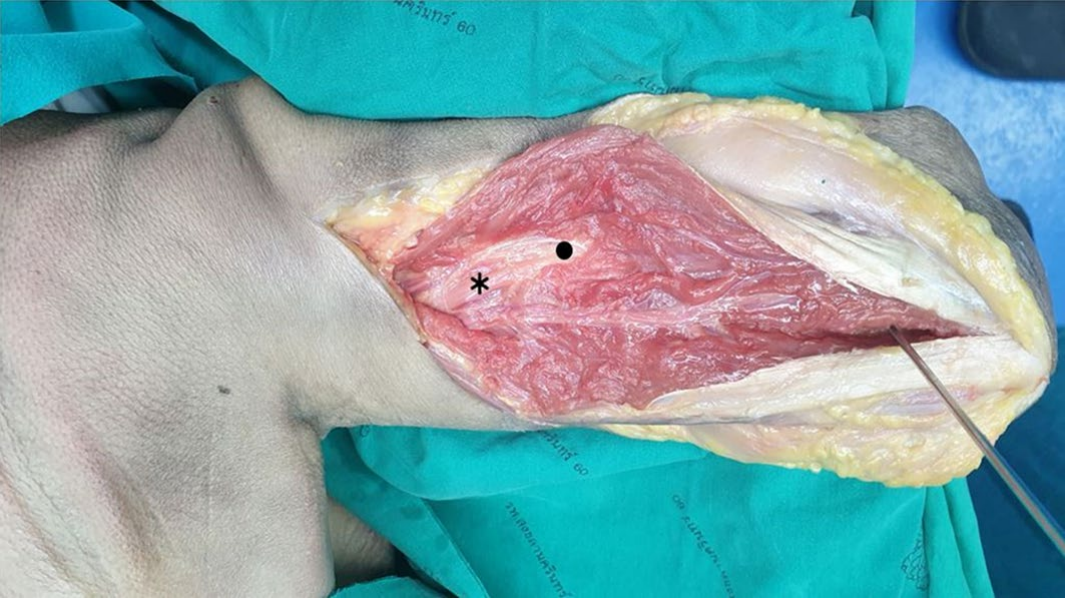
Illustration of the posterior approach via the triceps-split technique. *Center of the radial nerve in the posterior shaft of the humerus. • Radial nerve at the lateral intermuscular septum.

**Figure 3:**
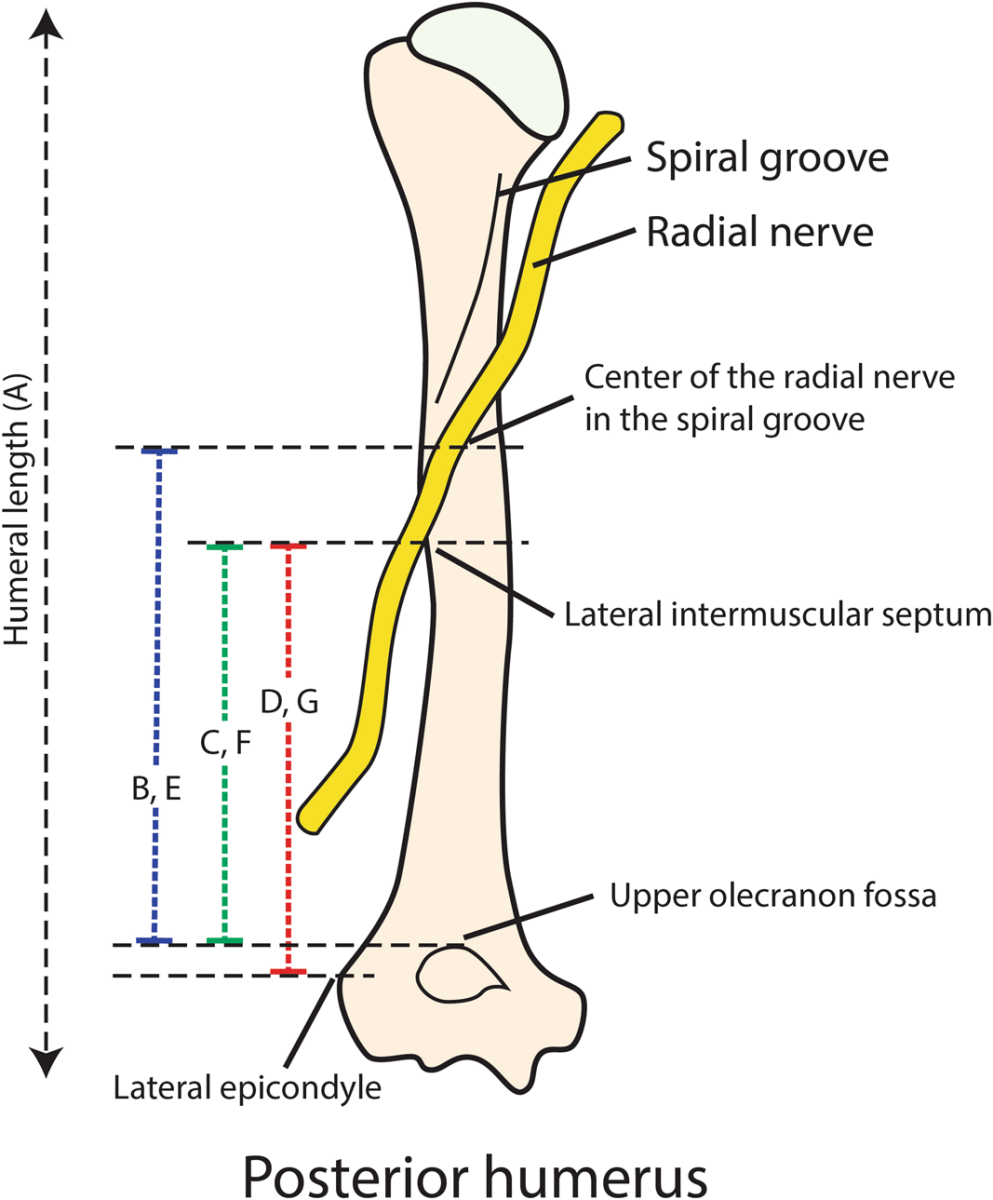
Illustration of the text references for each distance. A: Humerus length: posterior acromion to the tip of the olecranon. B: Upper olecranon fossa to the radial groove in the prone position. C: Upper olecranon fossa to the lateral intermuscular septum in the prone position. D: Lateral epicondyle to the lateral intermuscular septum in the prone position. E: Upper olecranon fossa to the radial groove in the lateral decubitus position. F: Upper olecranon fossa to the lateral intermuscular septum in the lateral decubitus position. G: Lateral epicondyle to the lateral intermuscular septum in the lateral decubitus position.

The upper part of the olecranon was marked with a K-wire as the anatomical landmark for the measurements. The distances from the upper olecranon to the radial nerve were measured with the cadaver in the prone position and at elbow flexion angles of 0°, 30°, 60°, 90° and 120° (B1, B2, B3, B4 and B5, respectively, in Fig. 2). Then, the point where the radial nerve passed laterally to the intermuscular septum was identified, and the distances from the upper olecranon to the radial nerve in the lateral intermuscular septum were measured using a digital Vernier caliper at elbow flexion angles of 0°, 30°, 60°, 90° and 120° (C1, C2, C3, C4 and C5, respectively, in Fig. 2). Then, the distance from the lateral epicondyle to the lateral muscular septum was measured at elbow flexion angles of 0°, 30°, 60°, 90° and 120° (D1, D2, D3, D4 and D5, respectively). Then, the cadaver was placed in the lateral decubitus position, and the distances from the upper olecranon to the center of the radial nerve in the radial groove (E1, E2, E3, E4 and E5), the lateral intermuscular septum from the upper olecranon (F1, F2, F3, F4 and F5) and the lateral epicondyle to the lateral muscular septum (G1, G2, G3, G4, and G5) were again measured at different elbow positions. The measurements of each of these distances are shown in Fig. 3. The elbow was held and adjusted by another surgeon to ensure the elbow position during measurement using a universal goniometer. Before moving the elbow to the next position, it was repositioned in the full elbow extension position. To decrease potential measurement bias, the distances were measured manually three times by two other experienced microsurgeons who did not dissect the cadavers.

### Statistical analysis

Each measurement is expressed as the mean ± SD. Statistical analysis was performed with the R program and the epicalc package (version 3.4.3). Differences between distances were compared using paired t-tests. A *P* value of 0.05 was considered significant in multiple comparisons.

## References

[1] Ekholm R, Adami J, Tidermark J, Hansson K, Törnkvist H, Ponzer S (2006). Fractures of the shaft of the humerus. An epidemiological study of 401 fractures. J. Bone Joint Surg. Br..

[2] Shao YC, Harwood P, Grotz MRW, Limb D, Giannoudis PV (2005). Radial nerve palsy associated with fractures of the shaft of the humerus: A systematic review. J. Bone Joint Surg. Br..

[3] Schwab TR, Stillhard PF, Schibli S, Furrer M, Sommer C (2018). Radial nerve palsy in humeral shaft fractures with internal fixation: Analysis of management and outcome. Eur. J. Trauma Emerg. Surg..

[4] Streufert BD, Eaford I, Sellers TR, Christensen JT, Maxson B, Infante A, Shah AR, Watson DT, Sanders RW, Mir HR (2020). Iatrogenic nerve palsy occurs with anterior and posterior approaches for humeral shaft fixation. J. Orthop. Trauma.

[5] Kamineni S, Ankem H, Patten DK (2009). Anatomic relationship of the radial nerve to the elbow joint: Clinical implications of safe pin placement. Clin. Anat..

[6] Hackl M, Damerow D, Leschinger T, Scaal M, Müller LP, Wegmann K (2015). Radial nerve location at the posterior aspect of the humerus: An anatomic study of 100 specimens. Arch. Orthop. Trauma Surg..

[7] Seigerman DA, Choung EW, Yoon RS, Lu M, Frank MA, Gaines LRJ (2012). Identification of the radial nerve during the posterior approach to the humerus: A cadaveric study. J. Orthop. Trauma.

[8] Chaudhry T, Noor S, Maher B, Bridger J (2010). The surgical anatomy of the radial nerve and the triceps aponeurosis. Clin. Anat..

[9] Simone JP, Streubel PN, Sánchez-Sotelo J, Steinmann SP, Adams JE (2019). Fingerbreadths rule in determining the safe zone of the radial nerve and posterior interosseous nerve for a lateral elbow approach: An anatomic study. J. Am. Acad. Orthop. Surg..

[10] Artico M, Telera S, Tiengo C, Stecco C, Macchi V, Porzionato A (2009). Surgical anatomy of the radial nerve at the elbow. Surg. Radiol. Anat..

[11] Guse TR, Ostrum RF (1995). The surgical anatomy of the radial nerve around the humerus. Clin. Orthop. Relat. Res..

[12] Jain RK, Champawat VS, Mandlecha P (2019). Danger zone of radial nerve in Indian population—A cadaveric study. J. Clin. Orthop. Trauma..

[13] Wright TW, Glowczewskie F, Cowin D, Wheeler DL (2005). Radial nerve excursion and strain at the elbow and wrist associated with upper-extremity motion. J. Hand Surg. Am..

[14] Chen WA, Luo TD, Wigton MD, Li Z (2018). Anatomical factors contributing to radial nerve excursion at the brachium: A cadaveric study. J. Hand Surg. Am..

[15] Hoppenfeld S, De Boer P, Buckley R, Thomas HA (2009). Surgical Exposures in Orthopaedics. The Anatomic Approach.

[16] Maresca A, Fantasia R, Cianforlini M, Giampaolini N, Cerbasi S, Pascarella R (2016). Distal-third diaphyseal fractures of the humerus: choice of approach and surgical treatment. Musculoskelet. Surg..

[17] Müller ME, Koch P, Nazarian S, Schatzker J (1990). Principles of the classification of fractures. The Comprehensive Classification of Fractures of Long Bones.

